# Facing the challenges in implementing sexual health guidelines for cancer survivors

**DOI:** 10.1093/jsxmed/qdaf322

**Published:** 2026-01-05

**Authors:** Natasha Gupta, Daniela Wittmann, Ted A Skolarus, Christian J Nelson, Stacy Loeb, John P Mulhall

**Affiliations:** Department of Urology, New York University Langone Health and Manhattan Veterans Affairs, New York, NY 10016, United States; Department of Population Health, New York University Langone Health and Manhattan Veterans Affairs, New York, NY 10016, United States; Department of Urology, University of Michigan, Ann Arbor, MI 48109, United States; Department of Surgery, Urology Section, University of Chicago, 5841 S Maryland Ave, Chicago, IL 60637, United States; Department of Psychiatry, Memorial Sloan Kettering Cancer Center, New York, NY 10065, United States; Department of Urology, New York University Langone Health and Manhattan Veterans Affairs, New York, NY 10016, United States; Department of Population Health, New York University Langone Health and Manhattan Veterans Affairs, New York, NY 10016, United States; Sexual & Reproductive Medicine Program, Urology Service, Memorial Sloan Kettering Cancer Center, New York, NY 10065, United States

**Keywords:** oncosexology, implementation science, clinical practice guidelines, cancer, survivorship

Sexual dysfunction is a common, persistent, and distressing sequela of cancer. This is due to both the psychological impacts of a cancer diagnosis and side effects of related procedures and treatments. Given that unresolved sexual problems can have devastating effects on the lives of patients and their partners, multiple organizations such as the National Comprehensive Cancer Network, American Cancer Society, American Society of Clinical Oncology, and American Urological Association provide guidance on diagnosis and treatment of sexual dysfunction in their cancer treatment guidelines. Recommendations include urging oncologists to refer patients to sexual medicine specialists and/or sex therapists (psycho-oncologists). However, studies demonstrate that sexual health frequently goes unaddressed in cancer care, and many patients and partners report unmet sexual health needs. This is unfortunate and surprising given the existence of multiple effective treatments for sexual dysfunction (psychotherapy, medications, surgery, and sexual aids).

These gaps in care are, in part, a manifestation of larger trends within medicine. Specifically, relegation of sexual health to the field’s margins combined with poor adherence to evidence-based guidelines in real-world practice settings. Indeed, a critical step in improving sexual health outcomes after cancer is to address widespread barriers to implementing sexual health clinical practice guidelines. Although there is a dearth of studies focused on this, studies in other areas have examined intrinsic and extrinsic factors leading to guideline-discordant care. A recent systematic metareview included 25 systematic reviews of barriers and facilitators of clinical practice guideline implementation across multiple specialties. Intrinsic barriers to guideline implementation included a lack of clarity and strength of evidence, while external barriers were time constraints, lack of provider knowledge, and inadequate organizational and leadership support.^1^ Overcoming these barriers is key to incorporating guideline recommendations into practice. Alternatively, facilitators of guideline implementation included presenting guidelines in a concise, user-friendly, and digital format to providers and patients; developing educational programs to improve provider knowledge; and having multidisciplinary teams, automated reminders, and support from administrators.^1^

Basing implementation strategies on frameworks such as the Knowledge-to-Action or Action, Actor, Context, Target, Time (AACTT) frameworks enables high degrees of specification for who needs to do what differently and in what contexts for guideline recommendations to be implemented in practice. Using implementation frameworks, models, and theories supports measurement of clinical effectiveness and implementation outcomes, while taking into consideration multilevel factors and rich clinical contexts contributing to implementation success or failure, all in support of generalizable knowledge.^2^ For example, the AACTT framework facilitates identification of specific behaviors (eg, intervention targets) that need to be changed to improve guideline adherence and factors influencing these behaviors across various providers and settings so strategies can be specific and, ideally, causally related to the outcomes.

With respect to sexual health, there are unique barriers to guideline implementation. One major barrier is the diffuse nature of sexual health recommendations for patients with cancer. Many recommendations are scattered across various sources and may be overlooked if they are buried within cancer site-specific guidelines covering all aspects of cancer care. Other sexual health recommendations are non-specific and contained within broader cancer survivorship guidelines applied to any patient with cancer. Given that each treatment and cancer type is associated with unique side effects, the former may not be adequately addressed. Finally, guidelines that focus on specific aspects of sexual dysfunction (eg, erectile dysfunction, testosterone deficiency, ejaculatory dysfunction) typically do not focus on patients with cancer. Therefore, although survivorship and sexual dysfunction guidelines both exist, they are unlikely to meet the needs of oncologists, patients with cancer, or partners.

To address these gaps for patients with prostate cancer (PCa), an international, multidisciplinary team of experts, supported by Movember™, developed the *Guidelines for Sexual Health Care for Prostate Cancer Patients* to create a comprehensive guide for providers, patients, and partners.^3^ These are the first guidelines focused on sexual health in a specific cancer. Key takeaways from these rigorous evidence and expert opinion-based guidelines include the application of a biopsychosocial model, the importance of pre-treatment sexual health counseling, the importance of pre-treatment assessment of sexual function and patients’ and partners’ sexual distress, routine and repeated assessment of the impact of diagnosis and treatment-related sexual side effects on the patient and the partner, counseling about the time course of sexual recovery/preservation across different treatments, moving focus away from solely penetration-based sexual activity, and being mindful of socio-ethnocultural variations in patients’/couples’ expectations and goals. The guidelines also include information about key topics such as testosterone deficiency and use of testosterone therapy, as well as recommendations for lifestyle modification to promote sexual health (eg, avoid smoking, engage in physical activity, eat more plant-based). Although these guidelines are specific to PCa, they contain generalizable sexual health principles that can serve as a model for other cancers.

While guideline generation is a key first step, barriers to implementation of guideline recommendations need to be identified, characterized within implementation frameworks, and targeted with evidence-based strategies to ultimately improve adoption and impact patient sexual health. One prominent barrier is poor patient–provider communication. Multiple studies demonstrate that patients and providers face barriers to discussing sexual health concerns in cancer care settings due to embarrassment, discomfort, societal norms, assumptions and biases, lack of provider training in sexual health, and time constraints.^4^ Since effective communication around this topic is central to addressing clinical care gaps, there is a critical need to evaluate the evidence base, determinants, and best practices associated with effective patient–provider communication to inform and tailor implementation interventions addressing gaps.

We acknowledge the above-mentioned barriers and proposed solutions are based on the available literature and our collective expertise. Therefore, there is a critical need to systematically evaluate implementation barriers facing sexual health guidelines across the breadth of providers, practice settings, and patient populations (eg, different marital status, sexual identity, etc.) to develop rigorous, multi-faceted interventions that ultimately improve guideline-concordant care and sexual health outcomes. Importantly, the aforementioned guidelines contain a host of recommendations, in this case 47, increasing the need for specificity in who needs to do what differently (eg, using the AACTT) for a given recommendation to be implemented. Perhaps focusing on the first one is an ideal place to start for population health: “*A clinician-initiated discussion should be conducted with the patient and the partner (if partnered and culturally appropriate), about realistic expectations of the impact of PCa therapy on the patient’s sexual function, the partner’s sexual experience, and the couples’ sexual relationship. The clinician should promote openness and inclusivity, consider cultural context, and tailor counseling to the specific needs of patients who are heterosexual, gay, bisexual (GBM), identify as men who have sex with men (MSM), transgender women, and gender non-conforming individuals. (Strong Recommendation; Evidence Strength Grade C)*” (Supplementary Table S1).

A next step might be to conduct an audit to understand the extent to which these discussions are happening using automated abstraction from electronic medical records, followed by an evidence-based, action-oriented feedback implementation strategy targeting clinician populations largely failing to enact the recommendation.^2^ Then, assessing the impact of the strategy on whether or not the discussions occur would not only address gaps in implementation and potentially improve sexual health outcomes for PCa, but also provide a rigorous framework as a model for other cancer survivor populations to help ensure sexual health issues are no longer marginalized.

## Supplementary Material

qdaf322_Supplementary_Table_1
