# Supplementary material for: Facing the challenges in implementing sexual health guidelines for cancer survivors
Source: J Sex Med. 2026 Jan 5;23(1):qdaf322. doi: 10.1093/jsxmed/qdaf322 (PMC12805888; doi:10.1093/jsxmed/qdaf322)
Supplement: qdaf322_Supplementary_Table_1 [file qdaf322_supplementary_table_1.doc]

Supplementary Table 1. Example of Action, Actor, Context, Target, Time (AACTT) Framework^2^ applied to the first recommendation promoting clinician-initiated discussions about sexual health within the Guidelines for Sexual Health Care for Prostate Cancer Patients^3^.

| **AACTT domain** | **Example** |
| --- | --- |
| Action | Clinician-led discussion about the impact of prostate cancer on the sexual health of patients and partners |
| Actor | Prostate cancer providers (e.g., physicians, advanced practice providers, nurses, psychologists, social workers, sexual health specialists) |
| Context | Oncology or Survivorship visit – exam room with patients’ partner present (if partner is available and the patient agrees) |
| Target | Patient diagnosed with prostate cancer and their partner (if partner is available and the patient agrees) |
| Time | Prior to prostate cancer treatment and at each follow-up oncology or survivorship visit |

^2^Presseau J, McCleary N, Lorencatto F, Patey AM, Grimshaw JM, Francis JJ. Action, actor, context, target, time (AACTT): a framework for specifying behaviour. Implement Sci. 2019 Dec 5;14(1):102. doi: 10.1186/s13012-019-0951-x. PMID: 31806037; PMCID: PMC6896730.

^3^Wittmann D, Mehta A, McCaughan E, Faraday M, Duby A, Matthew A, Incrocci L, Burnett A, Nelson CJ, Elliott S, Koontz BF, Bober SL, McLeod D, Capogrosso P, Yap T, Higano C, Loeb S, Capellari E, Glodé M, Goltz H, Howell D, Kirby M, Bennett N, Trost L, Odiyo Ouma P, Wang R, Salter C, Skolarus TA, McPhail J, McPhail S, Brandon J, Northouse LL, Paich K, Pollack CE, Shifferd J, Erickson K, Mulhall JP. Guidelines for Sexual Health Care for Prostate Cancer Patients: Recommendations of an International Panel. J Sex Med. 2022 Nov;19(11):1655-1669. doi: 10.1016/j.jsxm.2022.08.197. Epub 2022 Oct 1. Erratum in: J Sex Med. 2023 Apr 27;20(5):704. doi: 10.1093/jsxmed/qdad014. PMID: 36192299.
